# Preparation and research of new black zirconia ceramics

**DOI:** 10.1038/s41598-024-53793-8

**Published:** 2024-02-08

**Authors:** Yuxuan Ding, Qingchun Wang

**Affiliations:** 1 School of Materials and Metallurgy, Inner Mongolia University of Science and Technology, Baotou, 014010 Inner Mongolia China; 2 Inner Mongolia Autonomous Region Key Laboratory of Advanced Ceramic Materials and Devices, Baotou, 014010 Inner Mongolia China

**Keywords:** Ceramics, Composites

## Abstract

The existing black zirconia has problems such as uneven color development, poor stability, expensive raw materials, and harm to the human body and the environment. In order to solve the above problems, this paper intends to use NiAl_2_O_4_, NiTiO_3_, Fe_2_O_3_ as chromophore, zirconia as a matrix, and a solid-phase method is used to prepare high-performance black zirconia ceramics. The method avoids the introduction of toxic elements, and at the same time, it is more economical in the selection of color-developing pigments. The experimental results show that black zirconia ceramics with uniform color, continuous adjustment and high temperature stability can be obtained. When the sintering temperature is lower than 1450 °C and the color material mixing ratio is 10 wt.%, the mechanical properties and optical properties of the obtained samples optimum, the overall reflectance of the sample is less than 10 wt.%, which meets the conditions for market application. These black ceramics can be widely used in high-tech fields such as mobile phone backplanes, photovoltaic industry and high-end decorative materials, and have broad application prospects.

## Introduction

Zirconia is a recognized inorganic non-metallic material with high temperature resistance, wear resistance, good self-lubricating properties and biocompatibility^1,2^. There are many preparation methods for zirconia, such as solid-phase method, liquid-phase method, etc. But due to the high difficulty of operation of some liquid phase methods and serious environmental pollution, for example, waste gas pollution. In the process of preparing powder by liquid phase method, chemical reactions may produce some harmful gases, such as carbon dioxide, sulfur dioxide, nitrogen oxides, etc. If these gases are not treated or handled improperly, they may cause pollution to the atmospheric environment. In addition, there is waste liquid pollution and solid waste pollution, waste liquid will be produced in the process of preparing powder by liquid phase method, such as the remaining raw materials, catalysts, washing liquid and so on. If the waste liquid is not treated or handled improperly, it may cause pollution to the water environment. Some solid wastes produced in the process of preparing powder, such as sediments, residues, waste catalysts. If these wastes are not treated or handled improperly, they may cause pollution to the soil environment. Therefore, in order to reduce environmental pollution, the solid-phase method is generally used for the production of zirconia in factories. Colored ceramics are also widely used in medicine, photocatalytic degradation^3^, decoration and solar absorbers^4^ due to their good mechanical properties^5^, biocompatibility and excellent optical properties. Besides, colored zirconia is also widely used in mobile phone backplanes and luxury watch dials due to its good mechanical properties, metallic luster and signal non-shielding. As of today, people have not stopped developing new colored zirconia ceramics^6^, and have developed white, green, purple^7^, blue and other colors^8^. However, a large part of the preparation formula of black zirconia will be the addition of toxic metal oxides^9^. Such as hexavalent chromium ions, which are extremely harmful to the human body. Moreover, the high-temperature color instability and uneven color of black zirconia ceramics also hinder the application range of black zirconia^10,11^. S.W^12^ combine the advantages of additive manufacturing (AM) with those of functionally graded materials (FGM) to the development of thermoplastic 3d printing technology for ceramic-based 4D components. Black and white zirconia components are additively manufactured and co-fired without defects. Two pairs of different black and white zirconia powders were used to prepare different thermoplastic suspensions. The process parameters of single material test component were studied, and the process parameters of multi-color zirconia additive manufacturing were adjusted. Gargori et al.^13^ used electroplating waste Cr/Ni/Cu as a black ceramic pigment, but the color performance was not satisfactory, in which the L^*^ value was < 40 and the a^*^ and b^*^ value showed a nonlinear distribution, which indicated the color effect is not bright black. Costa^14^ used the solid-state method and found that the sample obtained by forming spinel with Fe, Ni, and Cr as the main phase has the best color rendering effect, and these raw materials can usually be obtained by recycling industrial waste. The color performance of the sample is better. Jiang Feng et al.^15^ successfully developed a novel colorant using solid-state method, with Fe_2_O_3_ and manganese oxide as the coloring groups. Its L^*^ value is 23.19, but a^*^ and b^*^ values are large. However, as a cobalt-free colorant, this is undoubtedly innovative. Mengke Liu^16^ prepared cobalt-free black ceramic pigments from stainless steel steelmaking dust, and studied its coloring mechanism. When Fe:Cr:Mn:Ni = 1:1:1:1, it was sintered at 1125 °C. After 30 min, a sample with uniform and excellent performance was obtained, and the coloring performance of the color material was better than that of the cobalt-containing color material sold on the market. Li Zhiqiao^17^ prepared black ceramics using Fe_2_O_3_,Cr_2_O_3_, MnO and NiO as raw materials by solid phase method , the main phases of which are NiFe_2_O_4_,NiCr _2_ O _4_ and Ni [Mn_0.5_Cr_1.5_] O_4_, the color is pure black, and with the increase of the amount of pigment added, the black becomes brighter and has excellent coloring performance, the L^*^, a^*^ and b^*^ values of the prepared sample are 18.02, 0.20 and 0 respectively. D. Melo^18^ and others tried to replace part of Co in lanthanum cobaltate with Ca to reduce the cost. N. W. Solís et al.^19^ prepared 3Y-TZP/GO composites by using zirconium-GO mixture by spark plasma sintering. The simultaneous sintering and in-situ reduction of graphene oxide opens up a very interesting technical route for the preparation of such materials. The effect of graphene content on the electrical, mechanical and optical properties of the material was studied, and the nanostructured black zirconia was obtained. The above-mentioned preparation methods of black zirconia ceramics all have problems such as not being close enough to black or introducing toxic metal oxides and high cost. Therefore, it is urgent to develop an environmentally friendly and economical black pigment doped with non-toxic metal oxides.

In this study, nickel oxide was used instead of cobalt oxide to prepare blue NiAl_2_O_4_. The green color material synthesized by NiAl_2_O_4_ and NiTiO_3_ is mixed with iron oxide red to obtain black color material, which greatly improves the use value of black zirconia. And the application of the solid phase method has no requirements on the sintering atmosphere and pressure, and the preparation is simple^20,21^. Based on the above research results, continuously tunable black zirconia ceramics with good mechanical and optical properties, economical and environmental protection can be prepared. The black ceramics can be widely used in high-tech fields such as mobile phone backplanes, photovoltaic industry^22^ and high-end decorative materials, and have broad application prospects.

## Experiment

### Experimental materials and methods

**Figure 1:**
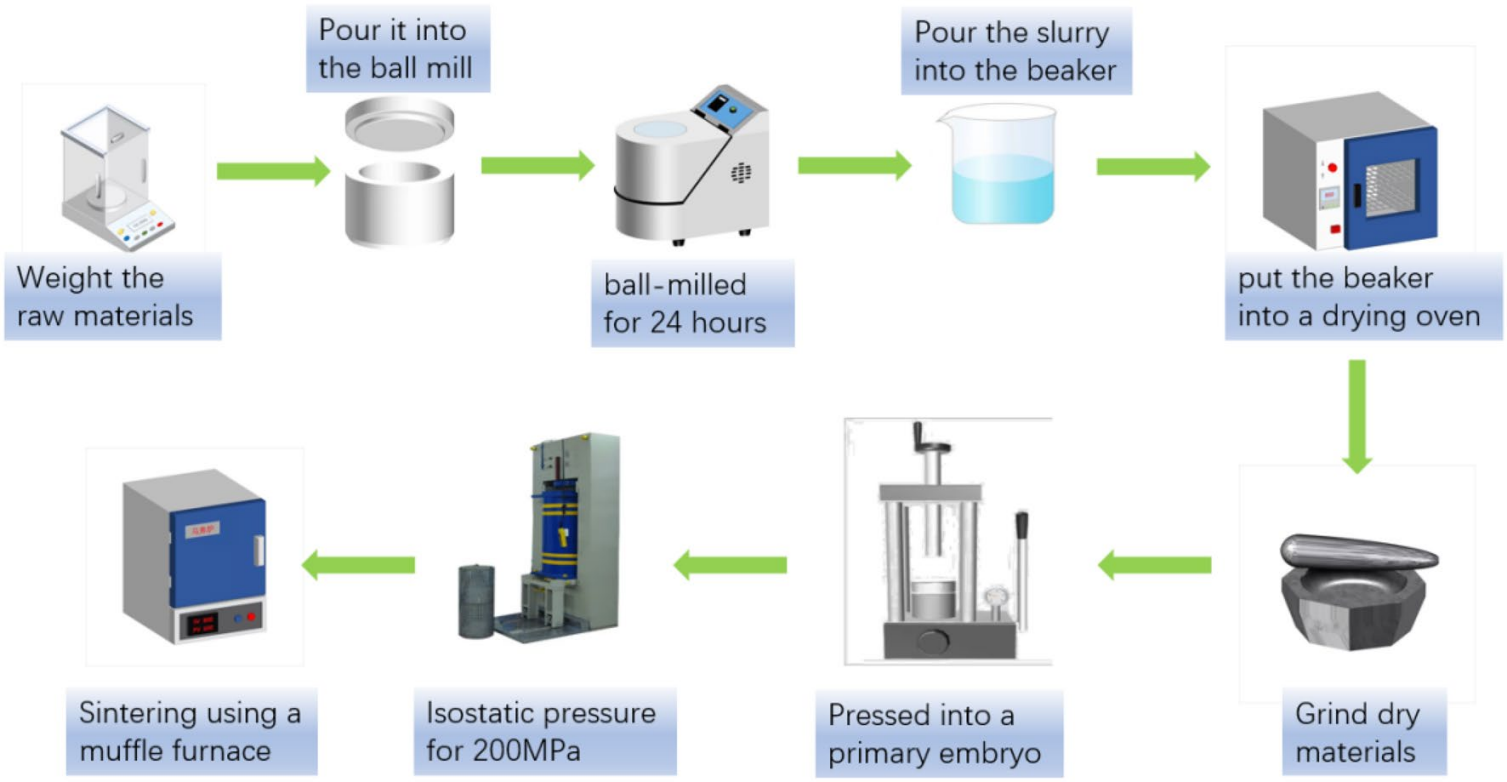
Preparation process of black zirconia.

In this paper, black zirconia ceramics were prepared by solid-phase method, with nano-scale zirconia stabilized by Y_2_ O_3_ as the matrix, and black zirconia ceramics were prepared according to the superposition principle of three primary colors of pigments. All raw materials used are shown in Table 1. First, prepare the NiAl_2_O_4_ precursor and the NiTiO_3_ precursor, mix TiO_2_, Al_2_O_3_ and NiO in proportion and perform ball milling for 24 h to obtain NiAl_2_O_4_ and NiTiO_3_, and then put them in a drying oven to dry. Afterwards, the prepared precursor and Fe_2_O_3_ were added to zirconia as a colorant, and after ball milling for 24 h, they were placed in a drying oven to dry, and the obtained agglomerated powder was ground and passed through a sieve to obtain a smaller particle size. Powder, by changing the doping amount of NiAl_2_O_4_, NiTiO_3_ and Fe_2_O_3_, ranging from 1 wt.%, 4 wt.%, 7 wt.%, 10 wt.%, and sintering temperature, to explore the influence on the performance of zirconia ceramics. Table 2 shows the mass percentage of raw materials. Figure 1 is preparation flowchart:

1. Weigh the raw materials according to the stoichiometric ratio and put them into the ball mill tank. The role of ball milling is to crush the raw material so that it meets the required granularity requirements. The purpose of adding anhydrous ethanol as a dispersant is to promote the dispersion and uniformity of the powder.
2. Ball mill with a planetary ball mill for 24 h, and then put the resulting slurry into a constant temperature drying oven at 80 °C to dry. The goal is to remove the alcohol and get the powder.
3. Grind the resulting powder with an agate mortar and sift it. The grinding process can change the shape and surface topography of the sample and increase the specific surface area of the material, thereby increasing the chance of interaction and reaction to obtain fine sample particles.
4. Press the obtained powder on the electric tablet press, and then press the obtained prepressed body at 200 MPa cold isostatic pressure. The aim is to reduce the distance between molecules without changing the appearance of the shape, thereby improving the physical properties of the substance.
5. The obtained embryo body is sintered at 1350 ℃, 1400 ℃, 1450 ℃, 1500 ℃, 1550 ℃ for 3 h, the purpose is to make the grain grow, the void is reduced, and the sample has better mechanical properties.

**Table 1:** Experimental material.

Chemical formula	Relative molecular mass	Purity	Manufacturer
TiO_2_	79.86	99.9%	Aladdin
NiO	74.71	99.9%	Aladdin
Al_2_O_3_	101.96	99.9%	Aladdin
ZrO_2_	123.22	99.9%	Aladdin
Fe_2_O_3_	159.69	99.99%	Aladdin
Y_2_O_3_	225.81	99.99%	Aladdin

**Table 2:** Experimental design of different additive amounts of colors.

NiTiO_3_ + NiAl_2_O_4_ + Fe_2_O_3_ (wt.%)	3YSZ (wt.%)	Sintering temperature (℃)
1	99	1350
4	96	1350
7	93	1350
10	90	1350

### Characterization methods

Details of all instruments are shown in Table 3. In this experiment, XRD was used to detect the phase of black zirconia, and the instrument produced by PANalytical company in the Netherlands was used. The scanning speed was 2°/min and the scanning range was 20°–70°. The phase information in the sample can be obtained by XRD, and then the material for further study, a metal copper block, is used as a diffraction target with a rated operating voltage of 40 kV and a rated current of 35 mA.

**Table 3:** Experimental instrument.

Name	Model	Manufacturer
Automatic pressure polishing machine	UNIPOL-1200S	Shenyang Kejing Automation Equipment Co., LTD
Automatic Vickers hardness tester	VIA-S	MATSUZAWA Corporation of Japan
Desktop electric tablet press	DY-20	Tianjin Keqi high-tech company
Electronic precision balance	YP2001N	Shanghai Precision Instrument Co., LTD
Ultrasonic cleaner	EQ-500E	Kunshan Ultrasonic Instrument Co., LTD
colorimeter	CM-3600A	Konica Minolta Investment Co. LTD
Cold isostatic pressing machine	DJY250/600	Taiyuan Zhongping Technology Co., LTD
X-Ray diffractometer	X’Pert Pro	PANalytical, Netherlands
Constant temperature blast drying oven	Model 101 electric heating	Shanghai—Heng Technology Co., LTD
Planetary ball mill	QM-3SP04	Nanjing University instrument factory
Ultraviolet spectrophotometer	UV-3900	Hitachi corporation
Scanning electron microscope	SIMGA-300	Carl Zeiss(AG) GMBH
Raman spectrometer	LabRAM HR Evolution	Horiba Scientific, Japan

The fracture and surface morphology of ceramic samples were observed by SIMGA-300 scanning electron microscopy. The samples were first treated with gold spraying for about 200 s to improve their electrical conductivity, and then placed in the sample chamber and vacuumed. Colorimetric measurement was carried out by CIE 1976 L^*^ a^*^ b^*^ colorimetric method.

In order to characterize the color change of the sample, the measurement was performed using a CM-3600A colorimeter. In a darkened room, the instrument is calibrated and the polished surface of the sample is wiped clean with an alcohol sponge. The samples were then tested with a UV-3900 UV–visible spectrophotometer. First, the pure white alumina dry pressed block is taken as the standard sample. After the instrument is calibrated, the complete ceramic sample sheet is polished to the surface without scratches by an automatic polishing machine, and the sample is scanned at a scanning rate of 2 nm/s to collect the reflectance between 200 and 800 nm, and the reflectance and reflection spectral range of the sample are measured.

Hardness characterization Vickers hardness is used to measure the relevant properties of a material. The HV-50 Vickers hardness tester is used to test polished ceramic sheets. The hardness test pressure is 5kgf and the holding time is 15 s. Hardness and toughness can be calculated by the calculation tool embedded in the test software, thus avoiding the error factor caused by manual calculation. The calculation methods of Vickers hardness and fracture toughness are as forum (1), (2):

1 $$ H_{\upsilon } = \frac{1.8554P}{{d^{2} }}, $$

2 $$ K_{IC} = 0.018\left( {\frac{E}{{H_{\upsilon } }}} \right)^{0.4} H_{\nu }^{\frac{1}{2}} \left( \frac{l}{a} \right)^{ - 0.5} . $$

## Results and discussion

### Phase analysis

**Figure 2:**
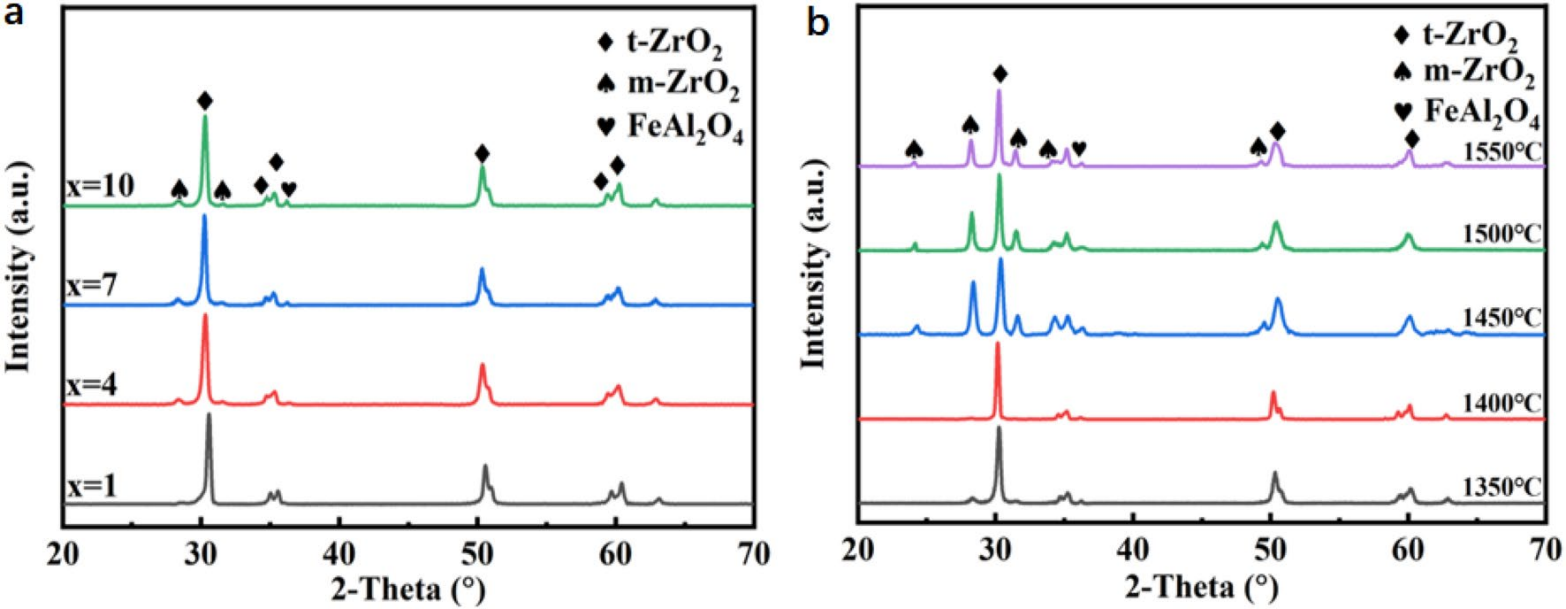
XRD pattern (**a**) of different colorant blending ratios, XRD pattern (**b**) of different sintering temperatures.

The crystal structure and phase of 3YSZ doped with NiAl_2_O_4_, NiTiO_3_, Fe_2_O_3_ as colorants were studied by XRD. The obtained XRD is shown in Fig. 2a (x is Fe_2_O_3_, NiAl_2_O_4_, NiTiO_3_ total mass fraction), it can be seen from the figure that different doping amounts have similar diffraction peaks, and when the colorant doping ratio is lower than 1wt.%, no peak of the FeAl_2_O_4_ is found in the XRD spectrum. The diffraction peaks indicate that the color material is basically solid-dissolved into zirconia at this time. As the color material mixing ratio increases to 7wt.% and above, it can be seen that there are monoclinic zirconia peaks and diffraction peaks of iron-aluminum spinel around 28° and 32°.This indicates that the amount of colorant that can be solid-dissolved in the zirconia matrix reaches a saturated value at this time. In the case of not changing the color material ratio, the sintered batch samples were sintered at 1350 °C, 1400 °C, 1450 °C, 1500 °C, and 1550 °C with a color material content ratio of 7 wt.%. From the obtained XRD Fig. 2b, it can be seen that with the increase of sintering temperature, the peak intensity of the diffraction peak of monoclinic zirconia in the sample gradually becomes stronger, which is due to the fact that when the sintering temperature is too high, the phenomenon of overburning occurs, and the appearance of the monoclinic phase may generally have a negative impact on the mechanical properties of the sample.

**Figure 3:**
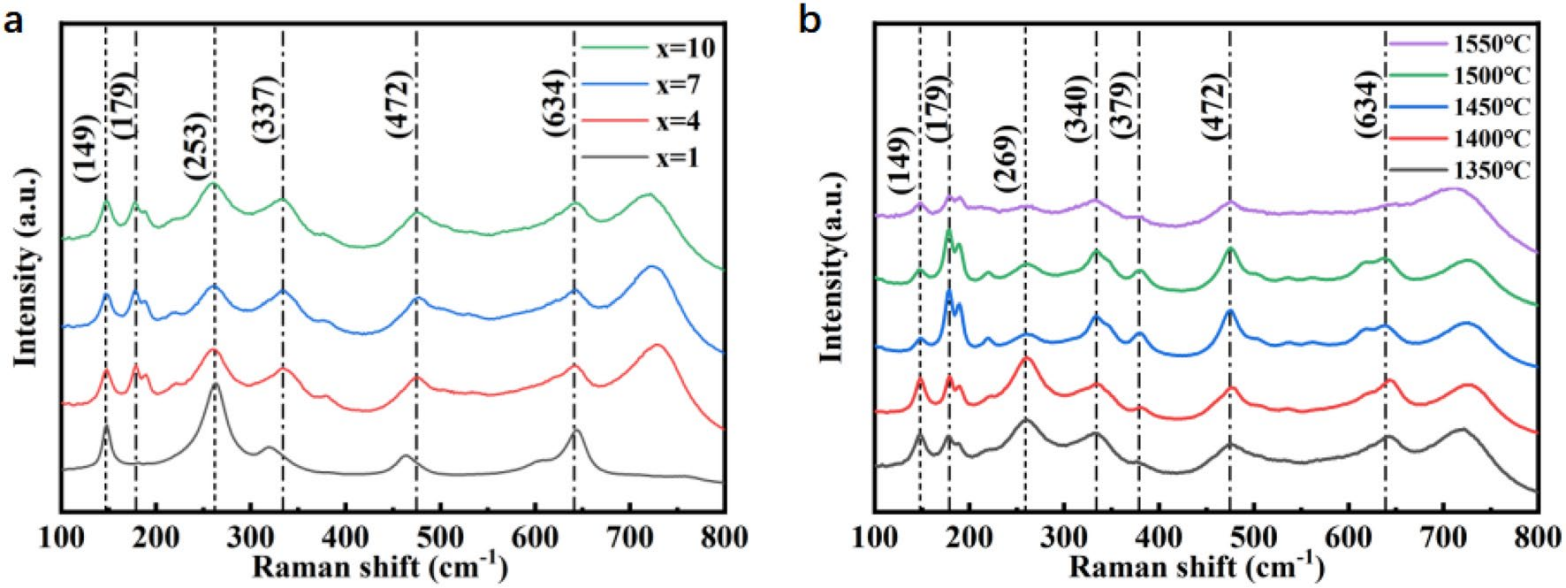
Raman pattern (**a**) of different colorant blending ratios, Raman pattern (**b**) of different sintering temperatures.

In order to further investigate the influence of color doping amount on the crystal vibration mode of the sample, Raman spectroscopy was used to analyze the sample. As can be seen from the Raman spectrum in Fig. 3a, when the color doping content is 1%, the sample is mainly tetragonal zirconia, which is marked by short lines in the figure. With the increase of doping amount of color material, in addition to tetragonal zirconia, monoclinic zirconia peaks also appeared in 179 cm^–1^, 337 cm^–1^, 472 cm^–1^ and 634 cm^–1^, which were marked by dot lines in the figure. At the position of 253 cm^–1^, the diffraction peak of the tetragonal phase begins to decrease, and the diffraction peak of the monoclinic phase increases, indicating that excessive doping amount of color material will affect the phase structure of the sample. As can be seen from Fig. 3b, when the sintering temperature rises to more than 1400 ℃, a monoclinic zirconia peak also appears at the position of 179 cm^–1^, and the intensity of diffraction peak is higher than that of tetragonal zirconia peak. This shows that the proportion of monoclinic phase in zirconia increases with the increase of sintering temperature.

### Microscopic morphology analysis

**Figure 4:**
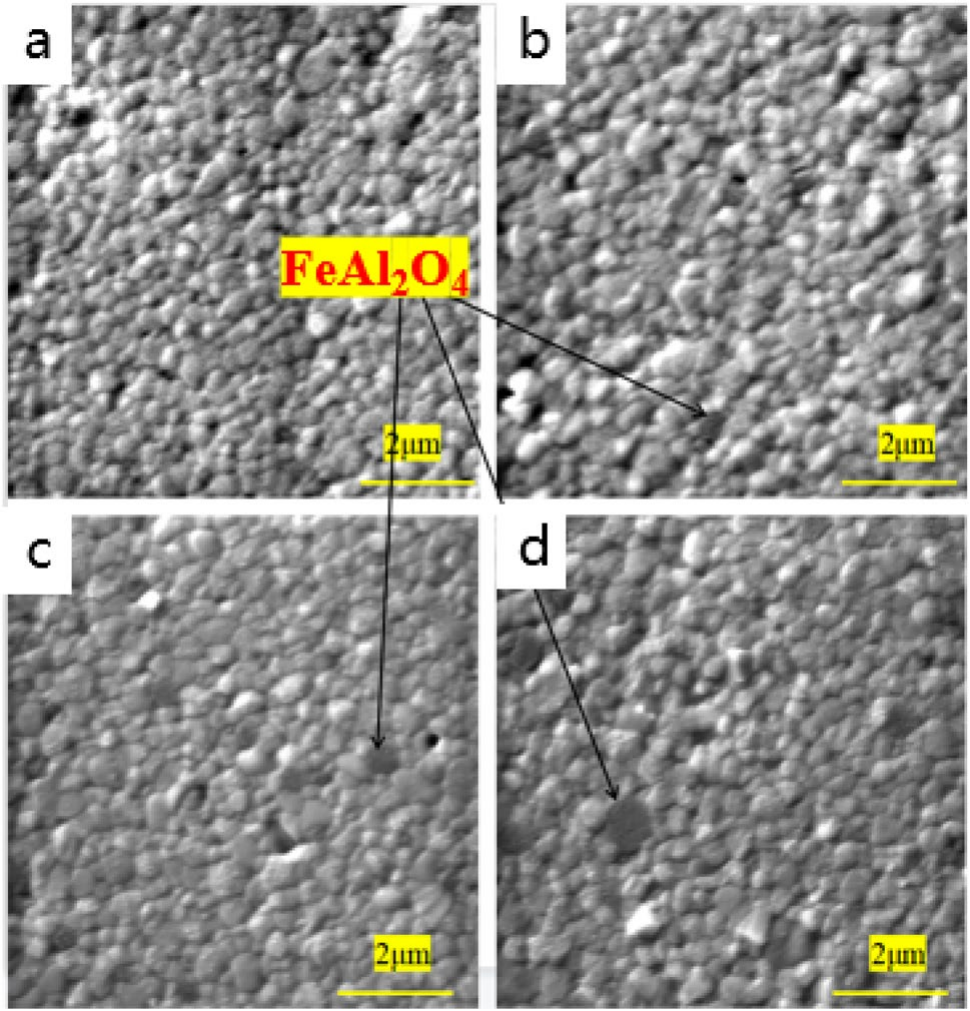
SEM images of cross-sections under different coloring ratios (x = 1, 4, 7, 10 wt.%).

**Figure 5:**
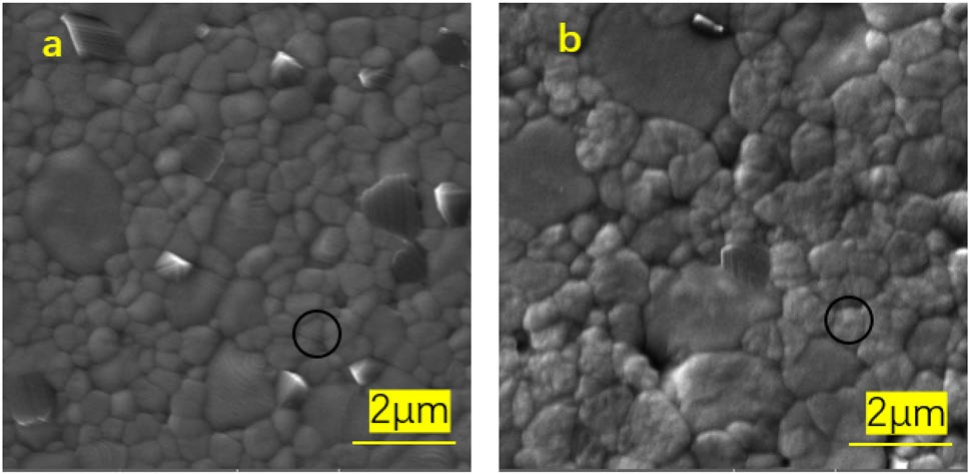
Surface SEM images at different sintering temperatures T = 1400 °C (**a**), T = 1450 °C (**b**).

SEM diagrams of ceramic samples at different sintering temperatures respectively, and a, b, c, and d in Fig. 4 are the microscopic appearance of samples with different doping amounts of colorants. It can be seen that the grain growth is relatively good, the size is uniform. By measuring the density of the sample using the drainage method, we found that the density of the sample is high, the density test results are shown in Table 4. And the grain shape in the figure is rounded for the zirconia grain. In the case of doping ratio, it has little effect on the grain size of the main phase. Figure 5a and b are the microscopic appearance of the sample surface at different sintering temperatures. Combining with the morphology under the electron microscope, it can be seen that as the sintering temperature increases, the crystal grains also become larger. As shown in Table 4, the grain size also increases with increasing temperature. When the temperature is 1450 ℃ and above, the grains grow abnormally, and the abnormally grown grains will affect the mechanical properties of the sample. First, the abnormally grown grains will reduce the volume of the surrounding grain boundaries, making the original thermal mismatch stress of zirconia grains and iron-aluminum spinel grains with different thermal expansion coefficients increases, which increases the internal stress of the sample, while the grains grow abnormally, exceeding the phase transition size, and a large number of tetragonal phases transform into monoclinic phase, the number of holes increases, which also leads to poor mechanical properties of the sample. This is consistent with the mechanical properties results obtained below.

**Table 4:** Grain size at different sintering temperatures.

Sintering temperature (℃)	Grain size (μm)
1350	0.33 ± 0.03
1400	0.39 ± 0.05
1450	0.46 ± 0.03
1500	0.49 ± 0.07

### Chromaticity and ultraviolet diffuse reflectance analysis

**Figure 6:**
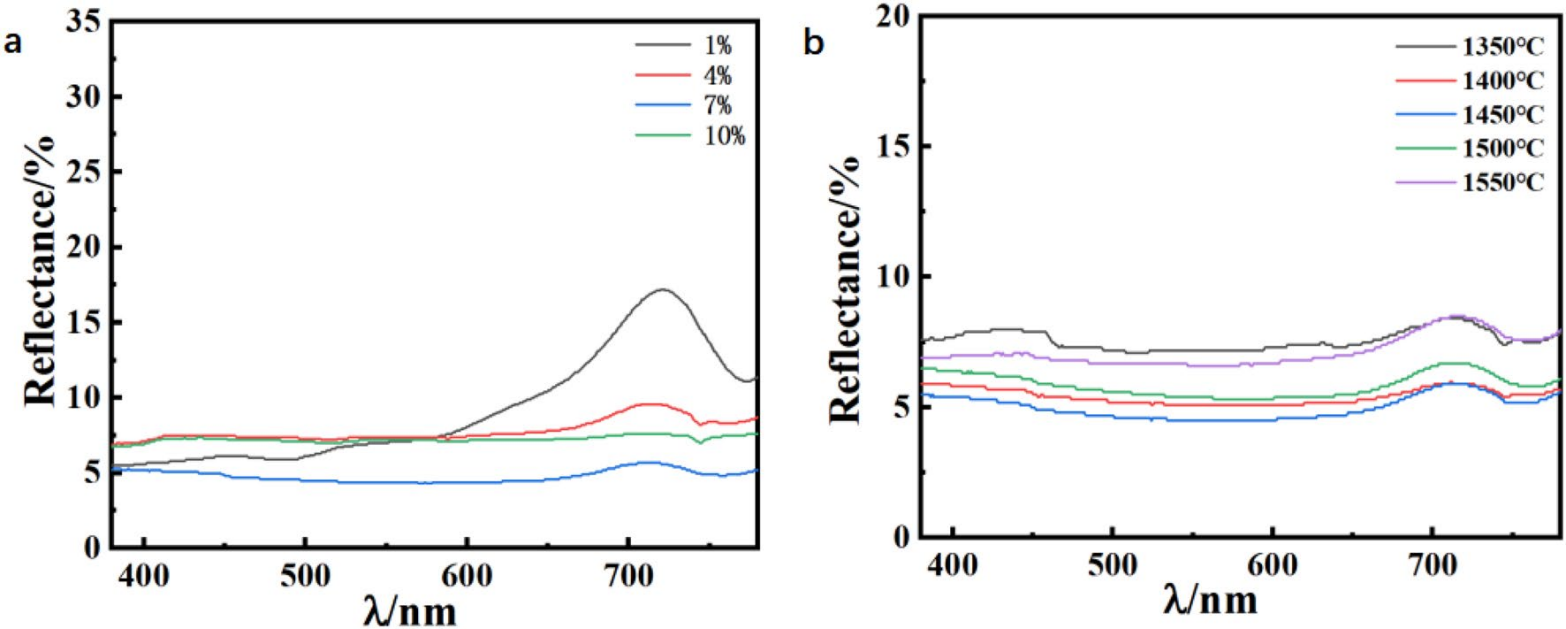
UV reflection diagram of different pigment blending ratios (**a**), UV reflection diagram of different sintering temperatures (**b**).

**Figure 7:**
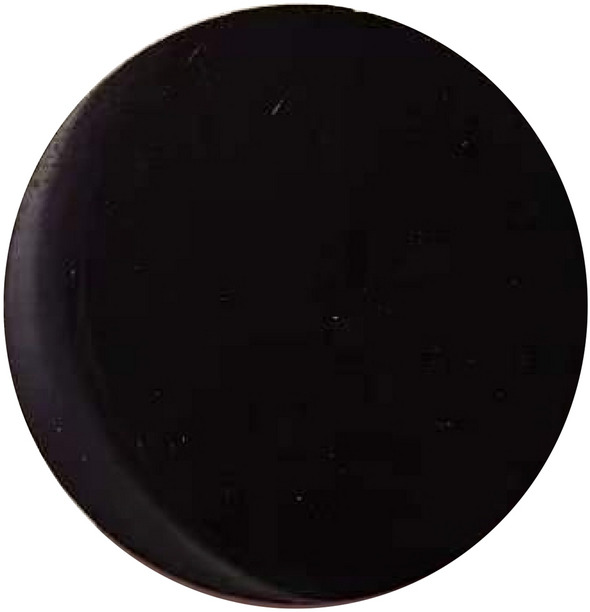
Sample actual picture.

As the data shown in Table 5, the brightness values of all the samples are greater than 40. And the a^*^ and b^*^ values of the samples are close to 0, except for the sample with 1 wt.% colorant doping. It can be concluded from the above data that the samples are bright black. As can be seen from Table 6, when the sintering temperature increases, the brightness value of the sample becomes higher, which is caused by a small amount of volatilization of the colorant at high temperatures. At this time the a^*^ and b^*^ values are still extremely close to 0, indicating that the sample at this time for the pure black color. Figure 6a and b are the UV reflectivity of samples with different amounts of pigment doping and different sintering temperatures, respectively. As can be seen from the Fig. 6a, except for the colorant doping of 1wt.%, the rest of the samples have no obvious reflection peaks. From the Fig. 6b, the reflectance of the sample did not produce a substantial change with the change in sintering temperature. Figure 7 shows the actual picture of the sample when the color doping content is 7 wt.% and the sintering temperature is 1350 ℃

**Table 5:** L*a*b* values of different colorant blending ratios.

Composition	L*	a*	b*
X = 1	47.3	3.31	5.22
X = 4	44.32	0.37	− 0.61
X = 7	44.26	0.38	− 0.60
X = 10	43.62	0.11	− 0.44

**Table 6:** L^*^a^*^b^*^ values of different sintering temperatures.

Temperature (℃)	L*	a*	b*
1350	44.26	0.38	− 0.60
1400	43.87	0.15	− 0.49
1450	44.01	0.44	− 0.23
1500	44.12	0.40	− 0.45
1550	45.20	0.39	− 0.68

### Analysis of mechanical properties

**Figure 8:**
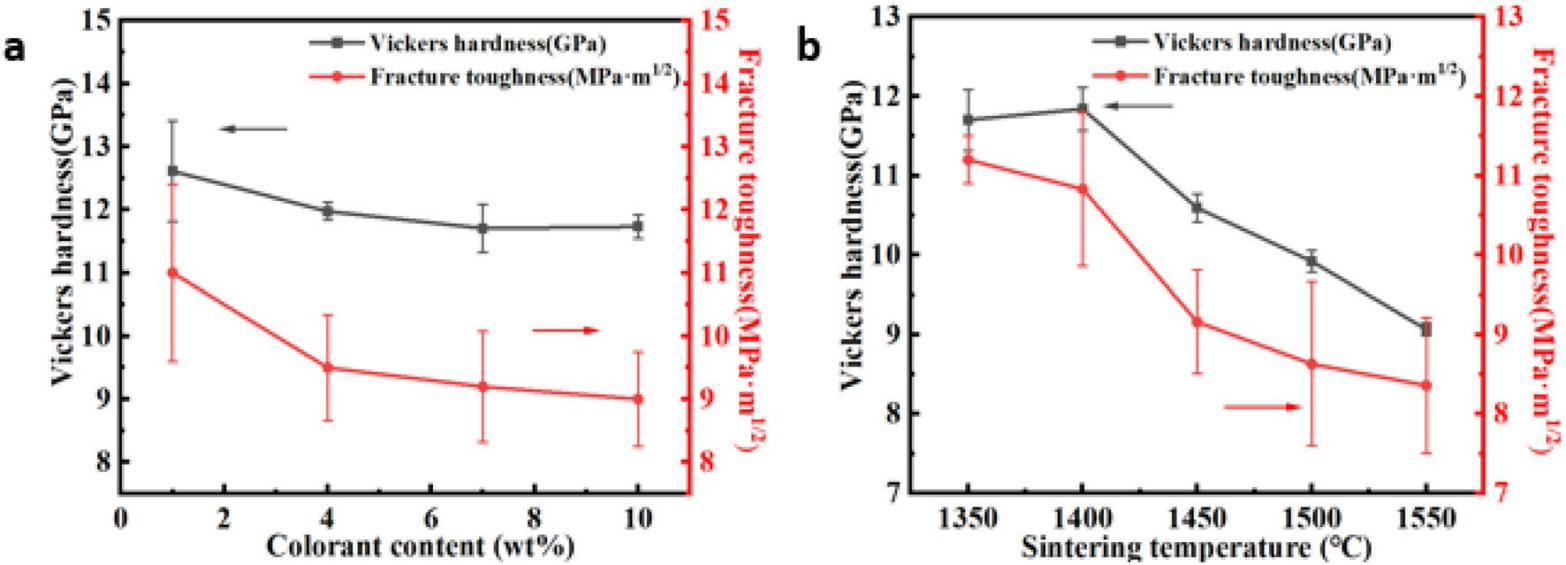
Vickers hardness and fracture toughness diagram of different pigment mixing ratios (**a**), Vickers hardness and fracture toughness diagram of different sintering temperatures (**b**).

Through the Vickers hardness and toughness calculation formula, the values of different pigment doping and different sintering temperatures can be obtained. From the overall trend of Fig. 8a and b, it can be seen that with the increase of pigment doping, the Vickers hardness and fracture toughness of the sample show a downward trend. The density of samples doped with different color materials was tested, and the results were shown in Table 7. The overall density of the sample showed a decreasing trend, which was consistent with the obtained test results of mechanical properties. It can be seen from Fig. 8a that the Vickers hardness and fracture toughness of the sample decrease with the addition of pigments. As dopants, the pigments reduce the original pure zirconia the mass fraction, and with the increase of the amount of pigment added, the pigment may also react with the stabilizer in zirconia, causing the sample to be unable to stabilize in the tetragonal phase, so it is mixed in ceramics based on zirconia too much will reduce the mechanical properties of zirconia ceramics, thus the mechanical properties of the samples show a downward trend. The lowest Vickers hardness is 11.73GPa, which can meet the requirements of commercial application. It can be seen from Fig. 8b that the Vickers hardness and fracture toughness also decrease with the increase of the sintering temperature. It can be seen from the combined XRD diffraction pattern that when the temperature rises, the content of zirconia in the monoclinic phase increases sharply. The change of phase structure of zirconia is often accompanied by the change of volume, which will increase the number of cavities inside the sample, resulting in the deterioration of the mechanical properties of zirconia, and the occurrence of overburning, which will cause a large change in the grain size. The internal stress becomes larger, so the mechanical properties become worse.

**Table 7:** Sample density value and relative density of different color doping amount.

Colorant content (wt.%)	Theoretical density (g/cm^3^)	Density (g/cm^3^)	Relative density (%)
1	6.11	6.03	98.6
4	6.11	6.01	98.3
7	6.12	5.97	97.5
10	6.08	5.94	97.6

## Conclusion

This paper reports a new method of preparing black zirconia ceramics without cobalt and non-toxic pigment doping. This sample exhibits enhanced visible light absorption and phase stability. The lightness L^*^ of the sample is about 43.62, and the a^*^ and b^*^ values are close to 0, all of which indicate that the sample is bright black. In addition, the color distribution of black ceramics is uniform and the color is stable. When the sintering temperature is 1400 ℃, the mechanical properties are the best, the highest can reach 12.6 GPa, and the reflectance of the samples are lower than 10 wt.%, so the samples prepared in this experiment meet the market requirements and have good application prospects.

## Data Availability

All data generated or analysed during this study are included in this published article.

## References

[1] Lv H, Bao J, Qi S (2019). Optical and mechanical properties of purple zirconia ceramics. J. Asian Ceram. Soc..

[2] Fanling Q, Zhipeng X, Jialin S (2011). Preparation of black zirconia ceramics by non-uniform precipitation method. J. Chin. Ceram. Soc..

[3] Teeparthi SR, Awin EW, Kumar R (2018). Dominating role of crystal structure over defect chemistry in black and white zirconia on visible light photocatalytic activity. Sci. Rep..

[4] Elisa Sani DS, Capiani C, Silvestroni L (2020). Colored zirconia with high absorbance and solar selectivity. Scr. Mater..

[5] Fokin PV, Solis Pinargote NW, Kuznetsova EV (2018). Effect of drying methods of alumina powder and graphene oxide mixture on the mechanical and electrical properties of sintered composites fabricated by spark plasma sintering. Inorg. Mater. Appl. Res..

[6] Liu HF, Wang H (2013). Current status and prospects of research on black ceramic colorants. China Ceram. Ind..

[7] Nataliia Gorodylova VK, Dohnalová Ž, Bělina P, Šulcová P (2013). New purple-blue ceramic pigments based on CoZr4(PO4)6. Dyes Pigments.

[8] Moya JS, Requena RMJ, Soria J (1988). Black color in partially stabilized zirconia. J. Am. Ceram. Soc..

[9] Chang Q, Wang X (2014). Encapsulated carbon black prepared by sol–gel-spraying: A new black ceramic pigment. J. Eur. Ceram. Soc..

[10] Liu, S. *et al.* Current situation and future prospects of research on black ceramic pigments. *Chin. Ceram. Ind. Mag.* **20**(6), 32–34 (2013).

[11] Xiang Z, Zhiqiao Li, Guojun Ma (2021). Research progress of cobalt-free spinel black ceramic pigments. Bull. Chin. Ceram. Soc..

[12] Steven Weingarten US, Johne R, Abel J, Schwarzer E, Moritz T, Michaelis A (2019). Multi-material ceramic-based components – additive manufacturing of blackand-white zirconia components by thermoplastic 3D-printing (CerAM - T3DP). J. Vis. Exp..

[13] Gargori C, Prim SR, Llusar M (2018). Recycling of Cr/Ni/Cu plating wastes as black ceramic pigments. Mater. Lett..

[14] Costa G, Della VP, Ribeiro MJ (2008). Synthesis of black ceramic pigments from secondary raw materials. Dyes Pigments.

[15] Jiang, F. *et al.* Preparation and coloring properties of new aluminum titanate-based cobalt-free black colorants. *Chin. Ceram.* **58**(11), 68–74 (2022).

[16] Mengke L, Guojun Ma, Xiang Z (2022). Preparation and coloring mechanism of cobalt-free black ceramic pigments from stainless steelmaking dust. Mater. Today Commun..

[17] Li Z, Zhang X, Ma G (2022). Preparation of Fe-Cr-Ni-Mn black ceramic pigment from stainless steelmaking dust. J. Chin. Ceram. Soc..

[18] Melo D, Vieira FTG, Costa TCC (2013). Lanthanum cobaltite black pigments with perovskite structure. Dyes Pigments.

[19] Solís NW, Peretyagin P, Torrecillas R (2017). Electrically conductor black zirconia ceramic by SPS using graphene oxide. J. Electroceram..

[20] Lv H, Bao J, Chao L (2019). Development mechanism of Ce-doped red zirconia ceramics prepared by a high-temperature reduction method. J. Alloys Compd..

[21] Lv H, Bao J, Ruan F, Zhou F, Wang Q, Zhang W (2020). Preparation and properties of black Ti-doped zirconia ceramics. J. Mater. Res. Technol..

[22] Elisa Sani ALMA, Jean-Louis Sans B, Sciti D (2015). Optical properties of black and white ZrO_2_ for solar receiver applications. Solar Energy Mater. Solar Cells.

